# Fast alignment-free sequence comparison using spaced-word frequencies

**DOI:** 10.1093/bioinformatics/btu177

**Published:** 2014-04-03

**Authors:** Chris-Andre Leimeister, Marcus Boden, Sebastian Horwege, Sebastian Lindner, Burkhard Morgenstern

**Affiliations:** ^1^Department of Bioinformatics, University of Göttingen, Institute of Microbiology and Genetics, 37073 Göttingen, Germany and ^2^Université d’Évry Val d’Essonne, Laboratoire Statistique et Génome, UMR CNRS 8071, USC INRA, 91037 Évry, France

## Abstract

**Motivation:** Alignment-free methods for sequence comparison are increasingly used for genome analysis and phylogeny reconstruction; they circumvent various difficulties of traditional alignment-based approaches. In particular, alignment-free methods are much faster than pairwise or multiple alignments. They are, however, less accurate than methods based on sequence alignment. Most alignment-free approaches work by comparing the word composition of sequences. A well-known problem with these methods is that neighbouring word matches are far from independent.

**Results:** To reduce the statistical dependency between adjacent word matches, we propose to use ‘spaced words’, defined by patterns of ‘match’ and ‘don’t care’ positions, for alignment-free sequence comparison. We describe a fast implementation of this approach using recursive hashing and bit operations, and we show that further improvements can be achieved by using *multiple* patterns instead of single patterns. To evaluate our approach, we use spaced-word frequencies as a basis for fast phylogeny reconstruction. Using real-world and simulated sequence data, we demonstrate that our multiple-pattern approach produces better phylogenies than approaches relying on contiguous words.

**Availability and implementation:** Our program is freely available at http://spaced.gobics.de/.

**Contact:** chris.leimeister@stud.uni-goettingen.de

**Supplementary information:** Supplementary data are available at *Bioinformatics* online.

## 1 INTRODUCTION

Traditional methods for comparative sequence analysis and phylogeny reconstruction rely on pairwise and multiple sequence alignments (Felsenstein, 2003). A major problem with these methods is that they are relatively slow because aligning two sequences takes time proportional to the product of their lengths. With the huge amount of sequence data that are produced by new sequencing technologies, faster methods for sequence comparison are required. *Alignment-free* methods became popular in recent years, as their runtime is usually proportional to the total sequence length; see Vinga and Almeida (2003) for a review.

Alignment-free methods are increasingly used for genome comparison, in particular for genome-based phylogeny reconstruction (Didier *et al.*, 2007; Hatje and Kollmar, 2012; Kolekar *et al.*, 2012), but also for fast protein clustering (Corel *et al.*, 2010; Hauser *et al.*, 2013; Lingner and Meinicke, 2006). These approaches are not only much faster than conventional alignment-based methods, but they also overcome some notorious difficulties in phylogenomics, such as finding ortholog genes (Ebersberger *et al.*, 2009; Schreiber *et al.*, 2009) or aligning large genomic sequences (Darling *et al.*, 2010). Other advantages of alignment-free genome comparison are that they can work with unassembled reads (Song *et al.*, 2012) and are not affected by genome rearrangements. Alignment-free methods are also used to construct *guide trees* for progressive multiple alignment (Blackshields *et al.*, 2010; Edgar, 2004; Katoh *et al.*, 2002). This could crucially improve the runtime of multiple-alignment algorithms, as calculating guide trees becomes the most time-consuming step in progressive alignment if the number of sequences grows.

Most alignment-free methods are based on *word frequencies*. For a fixed word length *k*, they calculate a (relative) word-frequency vector for each of the input sequences. Various distance measures on vector spaces can be used to calculate a pairwise distance matrix from these word-frequency vectors (Chor *et al.*, 2009; Höhl *et al.*, 2006; Sims *et al.*, 2009; Vinga *et al.*, 2012). Phylogenetic trees can then be calculated from these distance matrices with standard methods such as *UPGMA* (Sokal and Michener, 1958) or *NeighbourJoining* (Saitou and Nei, 1987). Other alignment-free approaches define the local *context* of sequence positions in terms of overlapping words containing a position (Didier, 1999). Some methods do not rely on a fixed word length *k* but allow for matches of variable length (Comin and Verzotto, 2012; Didier *et al.*, 2012; Haubold *et al.*, 2005; Ulitsky *et al.*, 2006). A common feature of all these methods is, however, that they are based on *exact* word matches between the input sequences.

*Database searching* is another traditional application of word matching in sequence analysis. Fast local alignment programs such as *BLAST* (Altschul *et al.*, 1990) originally relied on identifying word matches of a fixed length, so-called *seeds*. Rapid indexing methods can be used to identify such ‘seeds’, while, in a second phase of the algorithm, seeds are extended into both directions to find statistically relevant *high-scoring segment pairs*.

In a pioneering paper, Ma *et al.* (2002) proposed to use *spaced seeds* instead of *contiguous-*word matches as a first step in homology searching. Here, a fixed pattern or ‘mask’ of *match* and *don’t care* positions is defined, and two words of the corresponding length are considered to match if they coincide at the specified *match* positions, while mismatches are allowed at the *don’t care* positions. An obvious advantage of this approach is that word matches at nearby positions are statistically less dependent on each other than contiguous-word matches are. Also *spaced seeds* are better able to identify homologue sequence regions in the presence of mismatches. Ma *et al.* showed that *spaced seeds* are superior to contiguous-word matches in terms of *sensitivity* and *speed*; see also Brown (2008) for an overview.

Recently, we proposed to use *spaced words*, defined by patterns of *match* and *don’t care* positions, as a basis for alignment-free sequence comparison (Boden *et al.*, 2013). Instead of using spaced-word matches to trigger *local* alignments, we compare the spaced-word composition of sequences to define a measure of *global* similarity between them. In the present article, we describe an efficient algorithm based on *recursive hashing* and *bit operations* to calculate and compare spaced-word frequencies and we extend our approach to distance measures defined by *multiple* patterns. We use these distance measures to construct phylogenetic trees for real-world and simulated DNA and protein sequence families, and we compare our method to established alignment-free methods using *contiguous-*word frequencies, as well as to a traditional alignment-based approach. Our results show that, for phylogeny reconstruction, *spaced words* based on *multiple patterns* are superior to existing alignment-free methods that rely on *contiguous* words. A user-friendly web interface for our program is described by Horwege *et al.* (2014).

## 2 CALCULATING SPACED-WORD FREQUENCIES

As usual, for an alphabet Σ and 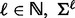 denotes the set of all sequences over Σ with length *ℓ*. For a sequence 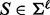 and 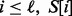 denotes the *i*-th character of *S*. Instead of ‘sequence’, we also use the term ‘word’ or ‘contiguous word’ to distinguish them from the *spaced words* that we are going to define. In our context, the alphabet Σ represents the set of nucleotides or amino acids, respectively. In analogy to the terminology introduced by Ma *et al.* (2002), we define for integers 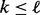 a *spaced word w* of length *ℓ* and *weight k* as a pair 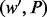 where 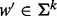 is a ‘contiguous word’ of length *k* and 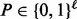 is a sequence of ‘0’ and ‘1’ characters of length *ℓ*, such that there are exactly *k* positions *i* in *P* with 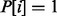. We call *P* the underlying *pattern* of *w*. In addition, we require that 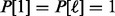 holds, i.e*.* the first and the last characters in *P* must be ‘1’. The ‘1’ positions in the pattern *P* denote *match* positions, while the ‘0’ positions are the *don’t care* positions.

Let 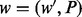 be a spaced word with weight *k* and length *ℓ* such that 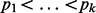 are the positions of the ‘1’ characters in *P*. We say that *w* occurs in a sequence *S* at position *i* if

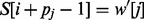

for all 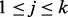. For example, the spaced word 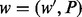 with 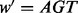 and P = 11001 occurs in the sequence 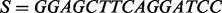 at positions 3 and 9.

To define a *distance function* on a set of input sequences 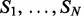 over Σ, we first consider a *single* fixed pattern *P* with weight *k*. For each sequence *S_i_*, we calculate the *relative frequencies* of all possible spaced words with respect to our pattern *P* (relative to the sequence length) and, similar as in other alignment-free approaches, each sequence *S_i_* is represented by the 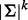-dimensional vector of these relative frequencies. It is then straightforward to define a *distance* 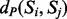 between two sequences *S_i_* and *S_j_* as the distance between these frequency vectors, using some standard distance metric on vector spaces.

This approach can be generalized by considering a whole *set*

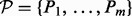

of patterns instead of a single pattern *P* [similarly, Li *et al.* (2003) used multiple spaced seeds for database searching]. Here, we define a distance 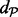 as the *average* of the distances defined by the patterns 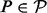, i.e. we define
(1)
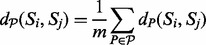



We call this extension the *multiple-pattern* version of our spaced-word approach.

## 3 IMPLEMENTATION

To calculate the frequencies of *spaced words* in a sequence with respect to a pattern *P*, we implemented a hash function that maps each spaced word to an integer in 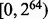. We first consider contiguous words of length *k* and define the *i*-th word of sequence *S* as 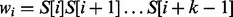. A fast way to hash successive words is by using a hash function *h* for which the value 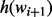 can be calculated in constant time from the previous value *h*(*w_i_*) by removing the value of the *i-th* character and adding the value of the new character at *i* + *k*. Such hash functions are called *recursive* or *rolling* (Cohen, 1997; Karp and Rabin, 1987). In practice, we used recursive hashing by cyclic polynomials (Cohen, 1997), which is also known as *Buzhashing* (Uzgalis, 1996).

While this approach is fast, it is possible, in principle, that *collisions* occur, i.e*.* that two different words are mapped to the same hash value. We do not correct such collisions but, instead, we define our hash function in such a way that the probability of such collisions is minimized. To this end, we use a hash function that provides a good uniformity for arbitrary input strings. First, we define a constant table *rtab* containing 256 values of 64-bit, assigning to each character of the alphabet Σ an integer in the interval 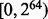. According to Uzgalis (1996), a uniform distribution of the resulting hash values is ensured by defining this array such that every vertical bit position has exactly 128 zeros and 128 ones. With this definition, collisions are extremely unlikely. In all test runs that we performed, we did not observe a single collision.

Next, we define the function *s* as the *barrel shift* that rotates the bits by one position to the left, *e.g.* we have 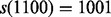. 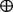 is denoted as the bitwise *exclusive or* operator. With these definitions, we define the hash value for a word *w_i_* as
(2)
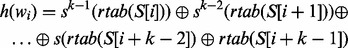



Once *h*(*w*_1_) is calculated with this formula, the values 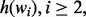 can be calculated recursively as
(3)
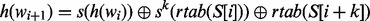



Note that, with this recursion, all hash values *h*(*w_i_*) can be calculated for a sequence *S* of length *n* in 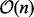 time, independently of the word length *k*. The advantage of using bit operations—compared with recursive hashing with multiplications and divisions—is that rotating bits is much faster than these algebraic operations.

So far, we only considered *contiguous* words. The above recursive formula (3) can be easily adapted to *spaced words* as long as the number of *don’t care positions* in the underlying pattern is small. To do this, we first calculate the hash value for words of length *ℓ*, and we then remove the terms corresponding to the *don’t care* positions in the underlying pattern. This corresponds to removing single characters from a word when a sliding window is moved to the next position. For example, if there is a single *don’t care* at position *p* in the pattern, we calculate 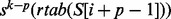 and apply the 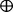 operator to erase the term corresponding to the character 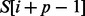 to calculate the hash value 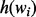 for the spaced word starting at position *i*. Consequently, the time complexity of this approach is 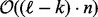 where *k* is the weight of the pattern *P*.

However, if the number of *don’t care* positions in *P* is greater than the number *k* of *match* positions, i.e*.* if 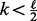, the recursive calculation is slower than the *naive* non-recursive approach, which calculates the hash values based on the match positions in the underlying pattern. In this case, the hash value *h*(*w_i_*) must be explicitly calculated for each spaced word *w_i_* leading to an 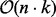 algorithm.

To store the frequencies of all *spaced words* in a sequence, we implemented a simple hash table. To determine an appropriate size of our hash table, we first calculate the maximum number of distinct (spaced) words. Clearly, for a sequence *S* and a pattern *P* of weight *k* and length *ℓ*, the maximum number of distinct spaced words in *S* is given by 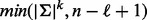 where *n* is the length of *S*. According to this observation, we choose as hash table size the smallest integer *b* such that 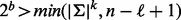. We then keep the *b* most significant bits of our 64-bit hash value, which is achieved by shifting the bits 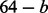 times to the right. These *b* bits are used as index in the hash table, resulting in a complexity of 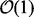 on average for the common operations such as search or insert. We handle collisions by sequentially searching the hash table for a free location, which is known as *open addressing*. This method led to a better performance in our tests than an alternative approach using linked lists.

Once the word frequencies are determined for our input sequences, we can easily compare them for different sequences, as a basis to calculate pairwise distances values. To do so, we iterate over both hash tables and for each key we search the equivalent key in the other hash table, which can be accomplished in 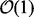 as mentioned above. If the key is not found in a hash table, then the corresponding spaced word does not occur in the other corresponding sequence.

In our *multiple-pattern* approach, we need to calculate spaced-word frequencies for a large number of patterns. To do this efficiently, we implemented *multithreading* in our program to increase the speed. Both steps, counting word frequencies and calculating pairwise distances are easily parallelizable. We are using threads to determine the word frequencies as well as for the computation of pairwise distances.

## 4 BENCHMARK SET-UP

To evaluate our approach and to compare it with other sequence-comparison methods, we used benchmark sequence sets from different sources: for DNA and protein sequence comparison, respectively, we used real-world as well as simulated sequence sets. Each sequence set consists of a number of evolutionarily related sequences together with a *reference* tree that we consider to be reliable.

For all sequences, we calculated the relative frequencies of *contiguous* as well as *spaced* words using the above-described *single-pattern* and *multiple-pattern* approaches. To obtain distance matrices for these sequence sets, we applied the *Jensen–Shannon (JS)* distance metric (Lin, 1991) to the obtained relative-frequency vectors. The *JS* distance between two frequency vectors *P* and *Q* is defined as
(4)
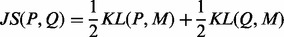

where
(5)
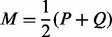

is the mean of *P* and *Q*, and *KL*(*P*,*M*) is the *Kullback–Leibler divergence* (Kullback, 1987) between *P* and *M* defined as
(6)
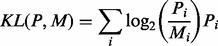

The JS metric was also used by Sims *et al.* (2009) for their *Feature Frequency Profile* (*FFP)* approach. In addition, we applied the *Euclidean* distance to the relative-frequency vectors obtained with our *m*ultiple-pattern approach.

Finally, we applied the *NeighbourJoining* program (Saitou and Nei, 1987) from the *PHYLIP* package (Felsenstein, 1989) to calculate unrooted trees from these distance matrices. As a comparison, we applied three state-of-the-art alignment-free approaches, *Average Common Substring* (*ACS*) (Ulitsky *et al.*, 2006), *K_r_* (Haubold *et al.*, 2005) and *FFP* (Sims *et al.*, 2009), as well as a traditional approach to phylogeny reconstruction using *Clustal W* (Thompson *et al.*, 1994) and *Maximum Likelihood* (Felsenstein, 1981). For *ACS*, we used our own implementation (Leimeister and Morgenstern, 2014) because the original software is not publicly available. For *FFP*, we used the word length that gave the best results for a given category of benchmark sequences. To evaluate these different methods, we compared for each sequence set the resulting trees with the corresponding reference tree using the *Robinson–Foulds* (*RF*) metric (Robinson and Foulds, 1981).

As test data for genomic sequence comparison, we used a set of 27 primate mitochondrial genomes that have been previously used by Haubold *et al.* (2009) to benchmark alignment-free methods. For these sequences, a reliable phylogenetic tree is known that is based on multiple sequence alignment and Maximum Likelihood. Further, we used a set of 13 fully sequenced flowering-plant genomes from the *Malvidae* clade plus the *grape vine* genome as outgroup. As a reference tree for these 14 genomes, we used a tree based on a multiple sequence alignment of manually assembled *CAP* and *Arp2/3* protein sequences as published by Hatje and Kollmar (2012). To benchmark our approach on protein sequences, we used *BAliBASE* 3.0, a standard benchmark database for multiple alignment (Thompson *et al.*, 2005). BAliBASE consists of 218 sets of related protein sequences together with *reference multiple alignments* based on 3D superposition that are considered to be reliable. As BAliBASE contains no information about the underlying phylogenetic trees, we constructed a reference tree for each sequence set by applying the standard *Maximum Likelihood* method (Felsenstein, 1981) to the corresponding *reference* multiple alignment.

In addition to these real-world benchmark sequences, we generated a large number of simulated genomic and protein sequence sets using the program *Rose* (Stoye *et al.*, 1998). This program mimics molecular evolution by producing a set of sequences along an evolutionary tree, starting with a common ancestral sequence at the root. Substitutions, insertions and deletions are randomly incorporated according to a pre-defined stochastic model of molecular evolution. As a result, sets of ‘evolutionarily’ related sequences are produced, together with known phylogenetic trees that we used as reference trees in our study. A parameter called *relatedness* determines the *average* evolutionary distance between the produced sequences, measured in *PAM* units (Dayhoff *et al.*, 1978). For DNA sequence comparison, we created a set of 50 sequences of 16 000 nt length each using *Rose* with a *relatedness* value of 70. To obtain simulated protein benchmark data, we generated a set of 125 protein sequences with a length of 300 *aa* each. Here, we used a *relatedness* value of 480. Except for the *relatedness* values, *Rose* was run with default values.

For all of these benchmark sequence sets, except for the 14 plant genomes, we first calculated trees based on *contiguous*-word frequencies, and we identified the word length *k* that produces the best results for the respective category of sequence sets, i.e*.* the trees with the smallest *RF* distances to the respective reference trees. For this value of *k*, we then generated patterns with weight *k*, i.e*.* with *k match* positions, and with up to 30 *don’t care* positions, i.e*.* with a length *ℓ* between *k* + 1 and *k* + 30. For each *ℓ*, we randomly selected a set 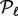 of 100 patterns of length *ℓ* and weight *k*. For small values of *ℓ* where <100 patterns are possible, we defined 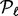 as the set of *all* possible patterns.

For each *ℓ* and each pattern 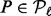, we applied our *single-pattern* approach and then calculated the *average RF* distances of the obtained trees to the respective reference trees. In addition, we applied our *multiple-pattern* approach by calculating the pairwise distance values 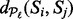 for each sequence set using *all* patterns 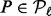 according to Equation (1). Moreover, we generated pattern sets 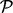 with 100 randomly selected patterns of weight *k* and with *varying* length *ℓ* for the *multiple-pattern* approach. We repeated these test runs using different sets of random patterns and calculated the standard deviations of the obtained *RD* distances to the reference trees. Note that this re-sampling is only possible if *ℓ* is large enough because for a short pattern length *ℓ*, the number of possible patterns is too small.

## 5 TEST RESULTS

### 5.1 Genomic sequences

**Fig. 1.**
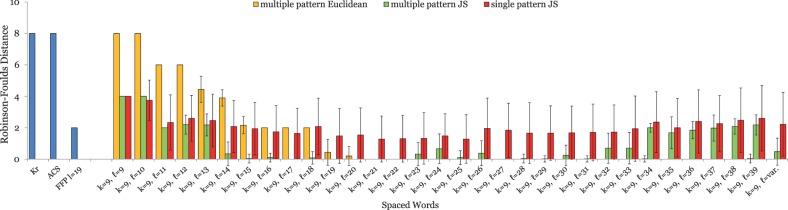
Test results on a set of mitochondrial genomes from 27 different primates; see Sections 4 and 5 for details. For a fixed pattern weight of *k* = 9, we generated patterns of length *ℓ* between 9 and 39, i.e*.* with 9 *match* positions and up to 30 *don’t care* positions. For each length *ℓ*, we randomly generated a set 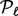 of 100 patterns—each pattern with *k* = 9 *match* positions and 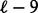 *don’t care* positions—and calculated pairwise distances (i) using all *single* patterns 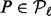 and (ii) using our *multiple-pattern* option where one distance value is calculated for a sequence pair using *all* patterns from 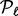. In addition, we used a set 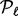 consisting of patterns with weight of *k* = 9, and with a *variable* number of *don’t care* positions (‘*ℓ* = var.’). We measured the *average RF* distances between the generated trees and the respective reference trees; the smaller these distances are, the better are the trees produced by a method. Where no green or yellow bar is visible, the *RF* distance is zero, i.e*.* here the tree topology calculated with the *multiple-pattern* approach exactly coincides with the reference topology. For the multiple-pattern approach, we repeated the test runs with 100 randomly selected sets of 100 patterns each and calculated the standard deviations of the resulting *RF* distances. For the single-pattern approach, we calculated the standard deviation for the 100 test runs with different patterns. Standard deviations are shown as error bars. Note that for *ℓ* = *k*, only one single pattern exists so here no standard deviations could be calculated. For small values of *ℓ*, <100 patterns are possible, so only a single set 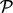 exists and standard deviations cannot be calculated for the multiple-pattern approach

For the primate mitochondrial genomes, the approach with contiguous words produced best results with a word length of *k* = 9, leading to a phylogeny with a *RF* distance of 4 to the reference tree. Thus, we generated patterns *P* with weight *k* = 9 and length *ℓ* between 9 and 39, i.e. with 9 ‘match’ positions and up to 30 ‘don’t care’ positions. The results are shown in Figure 1. For each pattern length *ℓ* > *k*, *spaced words* outperformed the standard *contiguous words* (*ℓ* = *k*). For practically all values of *ℓ*, the *multiple-pattern* approach with the *JS* distance led to better results than the *single*-pattern approach with the same pattern length *ℓ*. For some values of *ℓ*, the *RF* distance to the reference tree was 0, so here the tree topology reconstructed by our approach precisely coincides with the reference topology. For values *ℓ* < 19, the multiple-pattern approach with the *Euclidean* distance performed worse than with the *JS* distance and even worse than the single-pattern approach. For longer patterns, however, the Euclidean distance performed better; for *ℓ* > 20, multiple patterns with the Euclidean distance produced perfect tree topologies, i.e*.* the *RF* distance was 0. The established alignment-free approaches *K_r_* and *ACS* performed worse than spaced words with single or multiple patterns for all *ℓ* > 10. *FFP* performed better than these two approaches, but was outperformed by multiple spaced words with the *Euclidean* distance for all pattern lengths *ℓ* > 18 and by multiple spaced words with the *JS* distance for most values *ℓ* > 13.

The test results for our plant genomes are shown in Figure 3. Here, we used a set of 60 patterns of weight 14 and variable length.

**Fig. 2.**
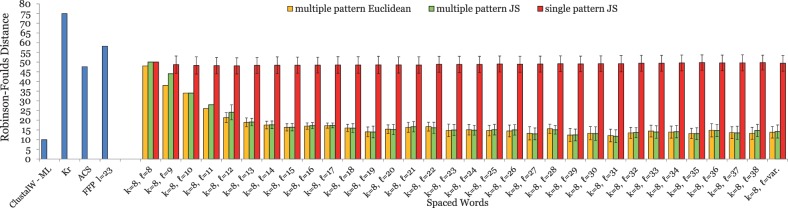
Test results on a set of 50 simulated DNA sequences of length 16 000 nt each. Patterns with a weight of *k* = 8 were used. Experimental conditions, notation and colour coding as in Figure 1. In addition to the various alignment-free methods, a classical approach using multiple alignment and *Maximum Likelihood* was used

For our simulated DNA sequences, we found that contiguous words with length *k* = 8 gave the best results, with an average *RF* distance of 50 to the respective reference trees. We therefore generated patterns with *weight k* = 8 and with length *ℓ* between 8 and 38. The results of these test runs are summarized in Figure 2. As can be seen, spaced words with *single* patterns and one or several *don’t care* positions (*ℓ* > *k*) performed better than the usual approach with *contiguous* words (*k* = 0), as long as the number of *don’t care* positions is small. The relative improvement in the quality of trees is modest, however, and if the number of *don’t care* positions is increased, the tree quality deteriorates. By contrast, a substantial improvement could be achieved by using our *multiple-pattern* approach. Here, increasing the pattern length *ℓ*—i.e. increasing the number of *don’t care* positions—further improved the resulting trees, leading to average *RF* distances of around 16 between the constructed trees and the reference trees. On these sequences, the difference between *JS* and *Euclidean* distance was small. The three competing alignment-free methods were clearly outperformed by our multiple-pattern approach. A classical approach using multiple sequence alignment and maximum likelihood led to slightly better results than our multiple-pattern program.

**Fig. 3.**
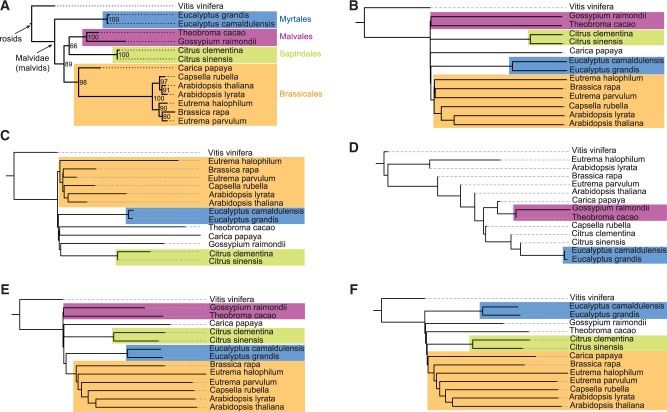
Trees reconstructed from 14 plant genomes: reference tree (**A**) based on aligned protein sequences and *Maximum Likelihood* from Hatje and Kollmar (2012) and trees reconstructed by the alignment-free methods *ACS* (**B**), *FFP* (**C**), *K_r_* (**D**), contiguous-word frequencies (**E**) with word length = 14 and *multiple spaced words* (**F**) with weight *k* = 14 using a set of *m* = 60 patterns. *RF* distances to the reference tree are *ACS:* 6, *FFS:* 10, *K_r_*: 18, contiguous words: 10, multiple spaced words: 6

### 5.2 Protein sequences

**Fig. 4.**
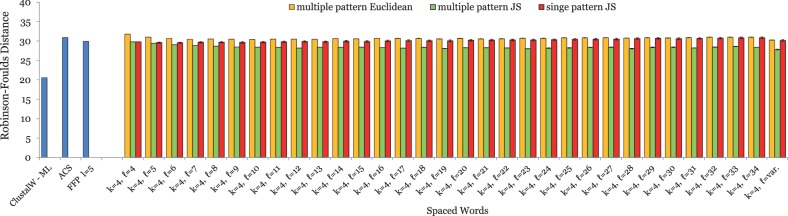
Test results on *BAliBASE*; the spaced-word approach was used with a pattern weight of *k* = 4. Notation as in Figure 1. For the multiple-pattern approach, 20 sets 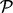 of patterns were generated, each set consisting of (up to) 100 patterns. Standard deviations of the *RF* distances for these 20 different pattern sets are shown as error bars for each value of *ℓ*. For the single-pattern version, 100 program runs with different patterns were performed and the standard deviation calculated. For the multiple-pattern option, standard deviations are so small that most error bars are not visible

Overall, our test results on proteins were similar to the results on DNA. On *BAliBASE*, a word length of *k* = 4 produced best results for *contiguous*-word frequencies. Thus, we used patterns with weight *k* = 4 and lengths *ℓ* between 4 and 34. Figure 4 shows the results of these test runs. Again, the spaced-words approach with *single* patterns gives better results than with contiguous words, but only for word lengths *ℓ* up to 7; a further increase in word length by using more *don’t care* positions led to deteriorated results, with *RF* distances to the reference trees larger than for contiguous-word frequencies. By contrast, the results of our *multiple* spaced-word approach led to better trees, and the quality of the trees further improved when the number of *don’t care* positions was increased. On these benchmark data, the results of our *multiple-pattern* approach were best when patterns of varying length were combined.

**Fig. 5.**
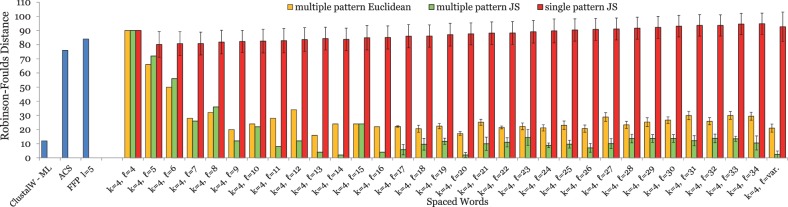
Test results on simulated protein sequences; the spaced-word approach was used with a pattern weight of *k* = 4. Notation as in Figure 1

Finally, the results of our test runs on simulated protein families are shown in Figure 5. Again, using distances based on *single* patterns improved the quality of the resulting trees compared with distances based on *contiguous*-word frequencies, but the improvement was relatively small and could only be achieved for small numbers of *don’t care* positions. Using distance measures combining different patterns led to far better results. As with the real-world protein sequence sets, the *multiple-pattern* approach with varying pattern lengths gave the best results. On our simulated protein families, this approach led to even better results than the classical approach based on multiple alignment and likelihood.

### 5.3 The number of patterns and program runtime

In the above test runs, we applied the multiple-pattern version of our approach with sets of *m* = 100 randomly selected patterns (*m* = 60 for the plant genomes) or used all possible patterns where fewer than *m* patterns are possible. The multiple-pattern option improved the performance of our method but is more expensive in terms of program runtime compared with the single-pattern version.

**Fig. 6.**
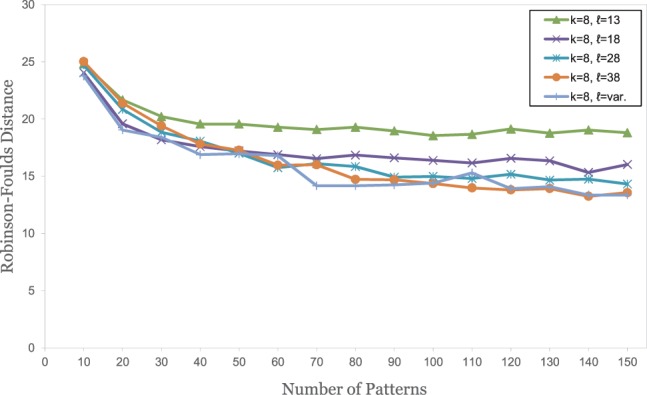
Influence of the number of patterns on the results of our *multiple-pattern* approach. The program was run on a set of 50 simulated DNA sequences of length 16 000 nt each, generated with *Rose*. For *k* = 8, different values of *ℓ* and *m* = 10,20, … ,150, we generated 100 sets 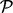 of patterns each, every set 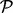 containing *m* patterns. The quality of the produced trees, measured as the average *RF* distance of the 100 trees to the respective reference trees, is plotted against *m*

**Fig. 7.**
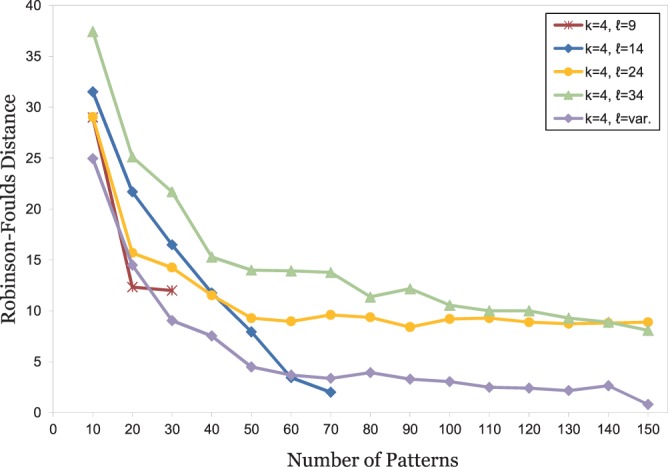
Influence of the number of patterns on the results of *Spaced words* applied to a set of 125 simulated *protein* sequences of length 300 *aa*, generated with *Rose*. Sets 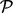 of patterns generated as in Figure 6. Note that for short pattern lengths *ℓ*, the set of all possible patterns is limited, so for *ℓ* = 9 and 14, only small values of *m* could be tested

To study the influence of the number *m* of patterns on the resulting trees, we used a set of 50 simulated DNA sequences of length 16 000 nt and a set of 125 simulated protein sequences of length 300 aa. We applied our multiple-pattern method to these datasets using different values of *k* and *ℓ* and pattern sets 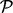 of different size *m*. The results of these tests are shown in Figures 6 and 7. As can be seen, the quality of the resulting trees improves if the number *m* of patterns is increased to 60 or 70, but a further increase in *m* does not lead to a significant improvement of tree quality.

To compare the runtime of our approach with other methods, we used two benchmark sets: a set of 50 simulated DNA sequences of 16 000 nt each and our test set of 14 plant genomes. In addition to the aforementioned programs for sequence comparison, we used *Clustal* Ω (Sievers *et al.*, 2011). All programs were run on an *Intel Core i7 4820 k* overclocked to 4.75 GHz. This CPU supports up to eight threads, which were fully used by our multithreading implementation. The results of these test runs are shown in Tables 1 and 2, respectively. *Clustal W* and *Clustal* Ω are omitted in Table 2 because they cannot be meaningfully applied to the whole genomes that we used.

**Table 1.** Runtimes of various established sequence-comparison methods and our spaced-word implementation on 50 simulated DNA sequences of length 16 000 nt each

Method	Runtime (s)
Clustal W	1817
Clustal Ω	1039
ACS	2.7
*K _r_*	0.9
FFP	123.3
Contiguous words	0.3
Spaced words, single pattern	0.31
Spaced words, multiple patterns	27.6

*Note*: Contiguous and spaced words were run with k = 8 match positions. The multiple-pattern option was used with sets 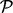 of 100 randomly selected patterns of varying length.

**Table 2.** Runtime on 14 plant genomes with a total size of 4.6 GB

Method	Runtime (s)
ACS	14 808
FFP	2123
*K_r_*	30,060
Contiguous words	207
Spaced words, single pattern	242
Spaced words, multiple patterns	20 295

*Note*: *Contiguous* and *spaced* words were run with *k* = 14 *match positions*. *Multiple-pattern* was run with 60 patterns of varying length.

### 5.4 Variation of sequence distances calculated with spaced words

**Fig. 8.**
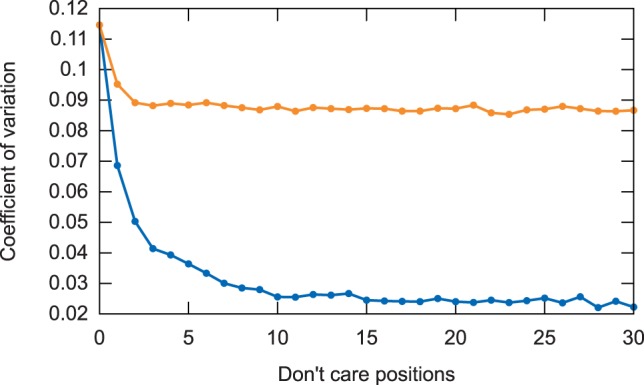
Variation coefficients of distance values calculated with *spaced-* and *contiguous*-word frequencies using *single* patterns (upper curve) and *multiple* patterns (lower curve) on 100 pairs of simulated DNA sequences; see subsection 5.4 for details. Note that for 0 *don’t care* positions, the spaced-word approach coincides with the contiguous-word approach (and the multi-pattern version coincides with the single-pattern version, as only one single pattern is possible for a given number *k* of *match* positions). Thus, for 0 *don’t care* positions, our graphic shows the variation coefficient for the classical *contiguous*-word approach

As mentioned, a main advantage of spaced-word frequencies is that occurrences of spaced words at different sequence positions are statistically less dependent on each other. Distance measures using spaced words can therefore be expected to be more stable. To study this point in detail, we generated 100 pairs of DNA sequences of length 16 000 nt with *Rose* and applied single and multiple *spaced words* to these sequences with *w* = 7 and *ℓ* between 7 and 37. We then calculated the *variation coefficients* of the resulting distance values. The results are summarized in Figure 8. As can be seen, the variation coefficients of the spaced-word distances are lower than for the standard exact-word approach (*k* = 0), and lower for the multiple-pattern option than for the single-pattern option.

## 6 DISCUSSION

Alignment-free methods are regularly used to estimate evolutionary distances between DNA and protein sequences and to construct phylogenetic trees. Most of these methods are based on word frequencies. Such approaches are usually less accurate than traditional phylogeny approaches that are based on multiple sequence alignments, but they are much faster. While aligning two sequences takes time proportional to the *product* of their lengths (Morgenstern, 2002; Needleman and Wunsch, 1970), word frequencies can be calculated in *linear* time. A certain disadvantage of word-based methods is the fact that word occurrences at neighbouring sequence positions are far from independent. For this reason, some authors proposed to correct word statistics by taking overlapping word matches into account (Göke *et al.*, 2012).

In database searching, word matches have been replaced by so-called *spaced seeds* where string matches according to a non-periodic pattern *P* of *match* and *don’t care* positions are used (Ma *et al.*, 2002). Motivated by this approach, we proposed to use *spaced words* instead of the traditionally used *contiguous words* to estimate distances between sequences and to construct phylogenetic trees. While, under an *i.i.d.* sequence model, the *expected* number of occurrences of a *spaced* word is approximately the same as for the corresponding *contiguous* word (obtained by removing the *don’t care* positions), spaced-word matches at neighbouring sequence positions are less dependent on each other if a non-periodic pattern *P* is used.

In the first version of this spaced-word approach, we used a tree structure to find matching spaced words (Boden *et al.*, 2013). Herein, we described an efficient algorithm and implementation based on *recursive hashing* and fast *bitwise* operations. While in general, algorithms using suffix-trees have the same linear time complexity as hashing algorithms, tree-based pattern searching is more difficult in our approach where patterns contain don’t care positions. Furthermore, the bitwise operations that we are using are much faster than operations on characters. The efficiency of our new approach enabled us to introduce a *multi-pattern* version where spaced-word frequencies with respect to an entire set 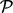 of patterns are used instead of a single pattern *P*.

Our test results show that *spaced-word* frequencies based on a *single* pattern with a small number of *don’t care* positions lead to better phylogenetic trees than *contiguous*-word frequencies, although the improvement that we could achieve with this first approach was limited; see also Boden *et al.* (2013). By contrast, we obtained a significant improvement by using our *multiple-pattern* approach. Here not only the resulting phylogenetic trees are superior to trees constructed with *contiguous*-word frequencies or *single-pattern* spaced words, but also the results are less sensitive to the number of *don’t care* positions. On simulated DNA and protein sequences, the *multiple-pattern* approach led to results not much worse—and sometimes even better—than the classical alignment-based approach to phylogeny reconstruction.

While the results of the *single*-pattern method strongly depend on the underlying pattern *P* (Boden *et al.*, 2013), our new *multiple-pattern* approach led to high-quality results with *randomly* selected sets 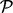 of patterns and the resulting trees are statistically more stable. However, the runtime of this approach is longer than if a single pattern is used, it is roughly proportional to the number of patterns in 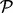. The *single-pattern* version of our approach is faster than all other programs that we tested, but the *multiple-pattern* version is slower than some state-of-the-art alignment-free methods. A program run on a set of 14 full-length plant genomes that cannot be handled by multiple-alignment methods took ∼4 min with the single-pattern version and about five and a half hours with the multiple-pattern version using a set 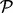 of 60 patterns.

A crucial parameter in our approach is the *weight k* of the patterns, i.e*.* the number of *match positions*. Clearly, optimal values for *k* depend on the length of the input sequences. In our study, we experimentally determined for a given group of sequences the optimal weight *k* for *contiguous* patterns and used this same value for our spaced patterns. First test results indicate that the optimal weight for contiguous-word matches also works best for spaced words (data not shown). There is no guarantee, however, that this is always the case, so further research is necessary to find suitable weights for *k* depending on the input sequences.

Another open question is which size *m* of the set of 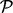 of patterns should be used. Figures 6 and 7 demonstrate that increasing *m* generally increases the quality of the resulting trees. For *m* > 60 or 70, however, no significant further improvement could be achieved in our test examples. It would be desirable to have a general rule to find a suitable number *m* of patterns in the multiple-pattern approach, depending on the input sequences. Finally, it is not clear which *distance* measure on the spaced-word frequency vectors is most suitable in our approach. In this study, we used the *JS* and *Euclidean* distance measures, but other distances may be more suitable to estimate phylogenetic distances between DNA or protein sequences based on their spaced-word frequencies.

To answer these questions, the statistical properties of spaced-word frequencies need to be analysed in detail, as has been done for the hit probabilities of *spaced seeds* in database searching (Keich *et al.*, 2004). Results on the probability of word occurrences (Robin *et al.*, 2005) may help to better understand the behaviour of our method theoretically, to optimize its parameters and to further improve its performance.

## Supplementary Material

Supplementary Data
