# Supplementary material for: Fast alignment-free sequence comparison using spaced-word frequencies
Source: Bioinformatics. 2014 Apr 3;30(14):1991–9. doi: 10.1093/bioinformatics/btu177 (PMC4080745; doi:10.1093/bioinformatics/btu177)
Supplement: Supplementary Data [file supp_30_14_1991__index.html]

Fast alignment-free sequence comparison using spaced-word frequencies — Fast alignment-free sequence comparison using spaced-word frequencies — Fast alignment-free sequence comparison using spaced-word frequencies — Supplementary Data 

# Fast alignment-free sequence comparison using spaced-word frequencies

## Supplementary Data

files

**Files in this Data Supplement:**

- Supplementary Data - pdf file
