# Supplementary material for: Fast alignment-free sequence comparison using spaced-word frequencies
Source: Bioinformatics. 2014 Apr 3;30(14):1991–9. doi: 10.1093/bioinformatics/btu177 (PMC4080745; doi:10.1093/bioinformatics/btu177)
Supplement: Supplementary Data [file supp_btu177_btu177spaced_supplementary.pdf]

Supplementary Material to

Fast alignment-free sequence comparison using spaced-word frequencies

Chris-André Leimeister, Marcus Boden, Sebastian Horwege, Sebastian Lindner, Burkhard Morgenstern

In our paper, we evaluate our *multiple-spaced-words* approach on various sets of benchmark sequences. For different values of k and ℓ, $k \leq \ell$, we generated sets $\mathcal{P}$ of *patterns* with weight k and length ℓ and calculted the spaced-word frequencies for the benchmark sequences with respect to these patterns as described in the paper. Distances between the sequences were calculated based on these spaced-word frequencies and *Neighbour-Joining* was applied to construct phylogenetic trees from the resulting distance matrices. Trees produced from spaced-word frequencies were compared to reference trees using the *Robinson-Foulds (RF)* metric.

In the paper, we repeated these test runs with different randomly generated sets $\mathcal{P}$ of patterns of the same weight k and length ℓ and calculated the standard deviations of the resulting *RF* distances; they are shown as error bars in Figures 1, 2, 4, 5 in the paper. For the sequence sets that we simulated with *Rose*, we also repeated our test runs with a single set $\mathcal{P}$ of patterns for each k and ℓ, but with 20 different sets of sequences. We then calculated the standard deviations for these different sets of input sequences. For DNA sequences, Figure 1 shows these standard deviations as error bars, the results for protein sequences are shown in Figure 2.

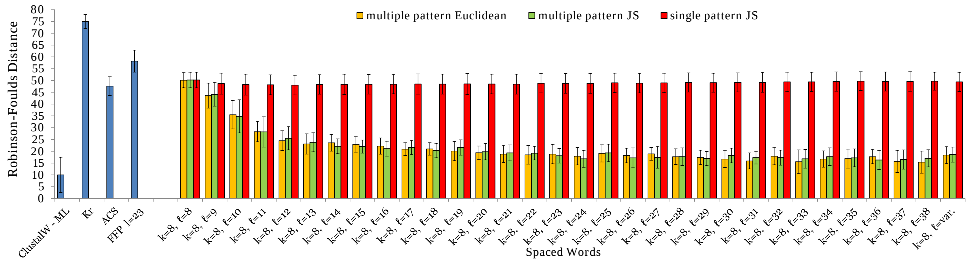

Figure 1: Test results on sets of DNA sequences generated with *Rose* for $k = 8$ and different values of $\ell \geq 8$. For each value of ℓ, 20 sets of DNA sequences were generated and, for the multiple-pattern option, the standard deviations of the *RF* distances to the respective reference trees are shown as error bars. For the single-pattern option, test runs with 100 patterns were performed and the standard deviations calculated.

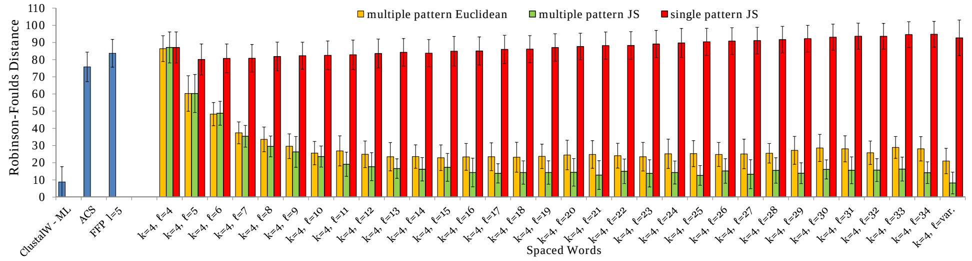

Figure 2: Test results on simulated protein sequences for patterns with a weight of $k = 8$. Standard deviations were calculated as in Figure 1.
